# Suitability of Single-Branched Thoracic Endografts for the Treatment of Acute Type B Aortic Dissection—An Anatomical Feasibility and Comparative Study

**DOI:** 10.3390/jcm15020558

**Published:** 2026-01-09

**Authors:** Julius Lang, Lorenz Meuli, Philip Dueppers, Alexander Zimmerman, Benedikt Reutersberg

**Affiliations:** 1 Department of Vascular Surgery, University Hospital Zurich, Rämistr. 100, 8091 Zurich, Switzerland; langjulius93@yahoo.de (J.L.); lorenz.meuli@usz.ch (L.M.); dr.dueppers@gmail.com (P.D.); alexander.zimmermann@usz.ch (A.Z.); 2 Department of Hand and Plastic Surgery, Spital Thurgau AG, 8501 Frauenfeld, Switzerland; 3 Department of Vascular Surgery, Kantonsspital St. Gallen, Rorschacher Strasse 95, 9007 St. Gallen, Switzerland

**Keywords:** single branch TEVAR, TEVAR, aortic dissections, endovascular aortic repair, Castor, TBE

## Abstract

**Objectives**: This study evaluated the anatomical suitability of two single-branched thoracic stent grafts—the Castor (Endovastec, China) and the Thoracic Branch Endoprosthesis (TBE, Gore, USA)—for proximal landing in aortic arch zone 2, including the left subclavian artery (LSA), in patients with acute type B aortic dissection (TBAD). While the TBE is currently available as an off-the-shelf device (26 main bodies, 8 branch configurations), the study also aimed to define the minimal number of configurations needed to treat most patients. The same approach was applied to the Castor stent graft, currently only available as a custom-made device (CMD), to assess its potential for off-the-shelf adaptation. **Methods:** A retrospective analysis was performed on computed tomographic angiographies of TBAD patients treated between 2004 and 2023. Exclusion criteria included type A or non-A-non-B dissections, isolated abdominal dissections, intramural hematomas, and lack of consent. Morphometric measurements were conducted using centerline analysis software. Suitability was defined per manufacturers’ criteria and reported with 95% confidence intervals. **Results:** Among 100 TBAD cases, 82% (95% CI: 73.3–88.3%) were suitable for the Castor CMD with 74 configurations. Main causes of exclusion were short landing zones and atypical arch anatomies. With adjunctive procedures, 13 Castor configurations covered all morphologies; 34% could be treated off-the-shelf, and 48% required additional interventions. For the TBE, off-the-shelf suitability was 22%, increasing to 78% with adjunctive procedures (six main bodies, five branches). **Conclusions**: Both stent grafts are promising for proximal extension in TBAD. Reduced configuration availability necessitates more adjunctive procedures, impacting efficiency and cost.

## 1. Introduction

Acute type B aortic dissection (TBAD) represents a potentially life-threatening condition requiring prompt and accurate diagnosis. In accordance with current recommendations, cardiovascular imaging plays a central role in the diagnostic work-up, with a multimodality approach integrating computed tomography, echocardiography, and magnetic resonance imaging to ensure comprehensive anatomical assessment and support treatment planning [1]. It is typically managed conservatively unless complications like impending aortic rupture, inadequate blood supply to peripheral or renal organs (malperfusion), treatment-resistant hypertension, or rapid expansion of the false lumen arise [2,3,4,5]. In such cases, and for prevention of long-term complications like aneurysmal degeneration of the false lumen, thoracic endovascular aortic repair (TEVAR) has become a minimally invasive and effective treatment approach [2,3,4,5]. The primary goal in TEVAR for TBAD is the closure of the primary entry tear. However, there is an ongoing debate about what length of proximal landing zone should be aimed for. It is known from the publication by Mesar and colleagues that in most cases of TBAD, the length of proximal healthy landing zone is <2 cm. Additionally, they showed that proximal landing in Ishimaru zone 2 is associated with a lower need for aortic reinterventions and aortic-related adverse events [6]. But to extent the proximal landing zone open or endovascular debranching of at least the LSA is necessary [7,8,9,10]. Open debranching is thereby associated with increased risks of stroke (8%), neck nerve injury (17%) or reintervention rates (8%) [11], underlining the need for endovascular solutions.

**Figure 1:**
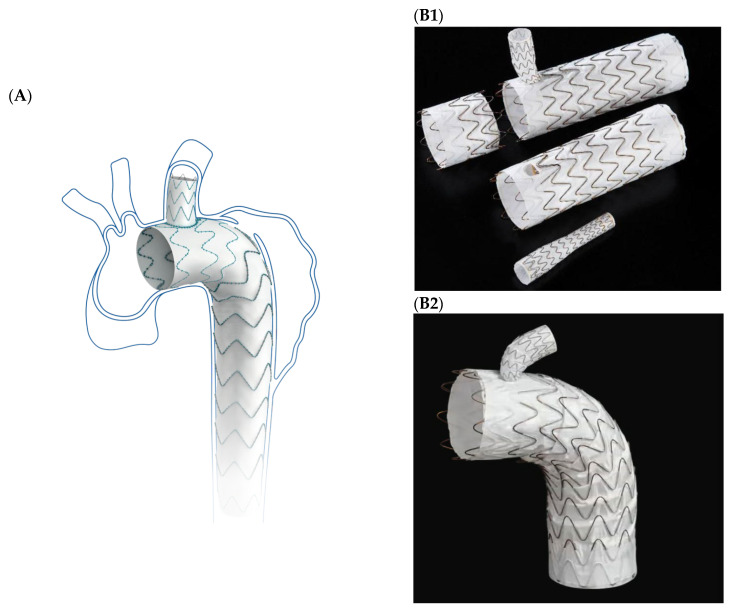
(**A**). Castor single-branched unibody stent graft (Endovastec, Shanghai, China)). (**B1**). Thoracic branch endoprosthesis (TBE) with branch and proximal extension (W.L. Gore & Associates, Inc., Flagstaff, AZ, USA). (**B2**). Branch in TBE-Branch-Portal.

Recently, two commercially available single-branched TEVAR devices were are on the Western market: the Castor stent graft (Endovastec, Shanghai, China) and the Thoracic Branch Endoprosthesis (TBE, W. L. Gore & Associates, Newark, NJ, USA) (see Figure 1) [12,13]. Castor is available as a custom-made device, whereby the TBE is already available off-the-shelf. Both are indicated to incorporate the LSA with landing in zone 2. This implicates that the proximal landing zone can be shorter overall and that the dislocation of the proximal thoracic stent graft into the LSA stump, which is commonly observed in practice after open LSA debranching, is made impossible.

So far, these endografts have only been examined individually in terms of their anatomical suitability in relatively small patient cohorts with mixed aortic pathologies. According to this data, morphological feasibility varied greatly, with only 28% in TBAD with the TBE and >65% for the Castor graft [13,14,15,16].

Overall, there is a lack of data from large cohorts consisting solely of patients with acute TBAD. Furthermore, the two prostheses have never been directly compared regarding their anatomical suitability. The aim of the study was therefor to analyze the anatomical suitability of the two commercially available single-branched thoracic stent grafts for landing in zone 2 in acute TBAD. In addition, a minimal number of off-the-shelf configurations should be identified that can be used to treat the maximal number of patients.

## 2. Patients and Methods

The presented retrospective observational study included all consecutive patients treated for acute (within 14 days after symptom onset) complicated or uncomplicated TBAD between 1 January 2004, and 31 December 2023, at a high-volume aortic referral center.

### 2.1. Inclusion Criteria

Patients were eligible for inclusion if they met all of the following criteria:

1. Diagnosis of acute Stanford type B aortic dissection (TBAD), defined as symptom onset ≤ 14 days before presentation.
2. Complicated or uncomplicated TBAD.
3. Availability of baseline computed tomography angiography (CTA) at presentation.
4. Provision of written informed consent for the use of clinical data.

### 2.2. Exclusion Criteria

Patients were excluded if any of the following conditions were present:

1. Stanford type A aortic dissection or non-A non-B aortic dissection.
2. Isolated abdominal aortic dissection.
3. Intramural hematoma without intimal tear.
4. Denied informed consent for data use.

Data on demographics, medical history, initial CTA findings, treatment strategy, and clinical follow-up were retrospectively extracted from the institutional medical information system.

The study was conducted in accordance with the Declaration of Helsinki and approved by the local ethics committee (Cantonal Ethics Committee Zurich, Switzerland; BASEC no. 2024-00303, approval date: 12 April 2024). Informed consent was obtained from all patients involved in the study.

### 2.3. Measurements

**Figure 2:**
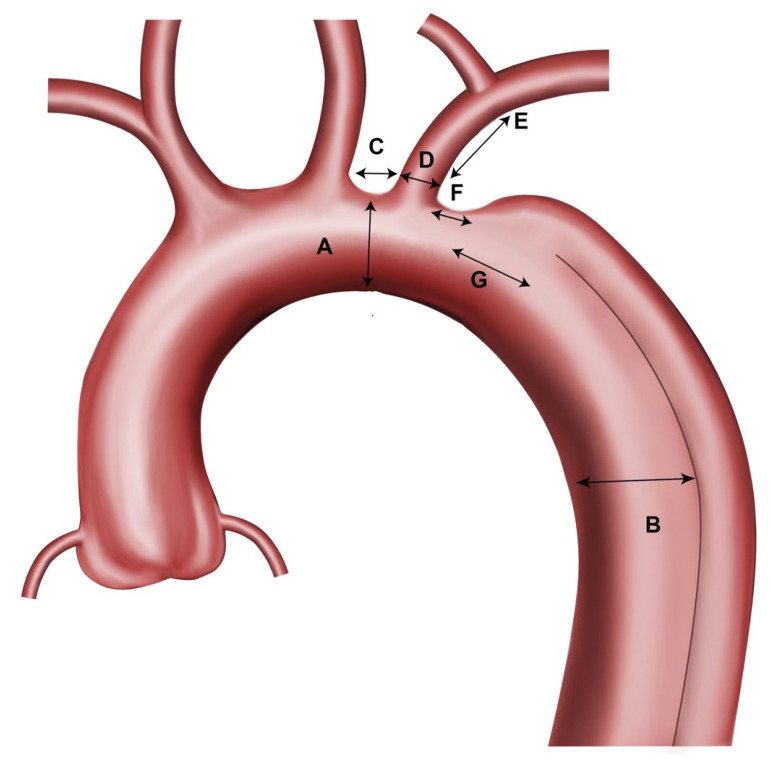
Measurement points.

The first CTA of each patient was assessed using a dedicated measurement software (EndoSize Version 3.2.1, Thereneva, Rennes, France). CTA assessment included aortic arch anatomy (length, diameters) and arch type, branch vessel anatomy (LSA length, diameter), and access vessel diameters to aid in selecting the appropriate grafts. The measurement points, measured in millimeter and from outer-to-outer wall, were (see also Figure 2):

- Maximum aortic diameter at proximal sealing zone (just distal to the left common carotid artery, within zone 2 of the arch) (**A**).
- Maximum aortic diameter at the distal sealing zone/estimated distal stent graft end (zone 4–5) (**B**).
- Covered stent graft length proximal to the branch ostium (outer curvature) (**C**).
- Ideal length of the stent graft to seal the primary entry tear.
- LSA diameter (**D**).
- LSA length (till the offspring of the first branch/vertebral artery) (**E**).
- Distance from the distal end of the LSA ostium to the beginning of the aortic dissection (**F**).
- Distance from the distal end of the LSA ostium to the beginning of the primary entry tear of the aortic dissection (**G**).
- Minimum left and right iliac artery diameter.
- Aortic arch type according to Marrocco-Trischitta et al. [17].

### 2.4. Device Descriptions

#### 2.4.1. Castor

The Castor single-branched stent graft (Figure 1A) from Endovastec (Shanghai, China) has been described in detail in several previous publications [13,18,19]. In brief, Castor consists of a self-expanding nitinol stent framework covered by woven polyester fabric. The delivery system consists of a hydrophilic outer 24-F sheath, as well as a soft polyester inner sheath which contains the stent graft main body and the incorporated side branch for the LSA (unibody design). The main body and the side-branch can be deployed separately. Currently, the Castor stent graft is currently only available as a custom-made device (CMD) with diameters ranging from 26 to 44 mm with 2 mm increments, and length from 6 to 21 cm. Combined with the configuration options for the LSA branch (diameter and length), more than 350,000 distinct configurations are theoretically available (Table 1) [13].

#### 2.4.2. Gore TAG Thoracic Branch Endoprosthesis (TBE)

The Gore AG Thoracic Branch Endoprosthesis (W.L. Gore & Associates, Inc., Flagstaff, AZ, USA, Figure 2 (A,B)), consists of a nitinol-based stent-frame woven into an expanded polytetrafluoroethylene fabric and has also been described previously [14,15,16]. In brief, the TBE is an off-the-shelf bi-modular device including a pre-cannulated main body and a dedicated side-branch. The main body has an 8 mm or a 12 mm inner-branch portal option and is available in lengths of 10, 15 or 20 cm depending on the diameter of the graft. The available main body diameters range from 21 to 45 mm. Overall, 26 different configurations are available for the main body. There is a total of eight different options available for the branch, with five for the 8 mm portal main body (with distal diameters ranging from 8 to 17 mm) and three for the 12 mm portal main body (with distal diameters ranging from 15 to 20 mm). A dedicated proximal aortic extender is available (Figure 2 (B) and Table 1).

For both prosthesis, please see Instructions for Use for complete device information, including approved indications and safety information.

### 2.5. Suitability Assessment

The optimal stent graft for each patient was selected based on the manufacturer’s anatomical recommendations, as determined by the morphological assessment (Table 2). In addition, the following factors were considered when selecting the appropriate stent grafts:

- Oversizing of the LSA by 1–2 mm.
- Minimum proximal landing zone length: 15 mm.
- Minimum LSA landing zone length: 15 mm.
- Oversizing of the main body proximally and distally of <10%.
- No covering of the ostium of the left common carotid artery (LCCA).
- For the TBE the length of the main aortic graft in dissection needs to be calculated: Distance **C + D + G + 10 cm** (Figure 2).

**Table 2:** The anatomic recommendations for patients with an aortic dissection according to the recommendations of the manufacture [13]. * minus the diameter of the LSA. LSA = left subclavian artery.

Parameter	Castor Single Branch Stent Graft	Thoracic Branch Endoprosthesis (TBE), 8 mm Portal	Thoracic Branch Endoprosthesis (TBE), 12 mm Portal
**Aortic diameter at the proximal sealing zone**	23–41 mm	16–42 mm	24–42
**Covered stent graft length proximal to the branch**	5–30 mm	≥15–20 mm *	33.5–36 mm *
**LSA diameter**	6–13 mm	6–15 mm	11–18 mm
**LSA length**	>25 mm	>25 mm	>25 mm
**Adequate access vessel caliber**	8 mm	7.5–9.5 mm	8.2–9.5 mm

Additional supra-aortic revascularization procedures, including carotid–subclavian bypass, chimney or parallel graft techniques, and proximal landing zone extension beyond zone 2, were not considered to achieve anatomical suitability. Patients requiring such procedures were classified as unsuitable.

For each patient meeting the anatomical suitability criteria, all potentially suitable graft configurations were identified for both platforms. The most frequently identified configurations for the overall cohort were then selected serving as “optimal stent grafts in stock”.

### 2.6. Proximal Sealing Length

The proximal sealing length for branched TEVAR devices is defined as the length from the proximal edge of the covered graft to the distal end of the LSA origin. For the TBE that is the covered proximal stent length up until the distal edge of the portal opening. For the Castor it is the proximal graft length up to the distal end of the branch origin.

### 2.7. Outcomes

The primary outcome was the anatomical suitability within the instructions for use of the respective stent graft. Anatomical suitability was reached if all anatomical requirements for the endografts were met and reported for the overall cohort. Additional supra-aortic debranching was not allowed to reach suitability.

The secondary outcomes included a comparison of the anatomical characteristics between patients for whom the stent graft implantation was feasible and those for whom it was not for each stent graft.

### 2.8. Statistical Methods

Continuous data were summarized with median and quartiles 1 and 3 and categorical variables with count and percentage. Suitability was reported as percentages with 95% binomial confidence intervals and compared between manufacturers using Fisher’s exact test.

Clinical, demographic, and anatomical characteristics were compared between patients for whom the stent graft implantation was feasible and those for whom it was not using the Mann–Whitney U test and Chi2 tests. All tests were two-sided and significant at an alpha-level of a 5%.

## 3. Results

### 3.1. Patient and Anatomic Characteristics

A total of 100 patients with acute TBAD met the inclusion criteria and was analyzed. The cohort had a median age of 63 years (q1–q3: 55.1–71.3 years) and was with 75% mainly male. Comorbidities and anatomical characteristics are shown in Table 3. CTA analysis showed that the median maximum proximal aortic diameter was 32 mm (30–34 mm), while the median maximum distal aortic diameter was 30 mm (26–31 mm). The median LSA diameter was 11 mm (9–12 mm). The median length from the left common carotid artery (LCCA) to the LSA was 10 mm (8–14 mm). The median distance from the LSA to the start of the dissection was 3 mm (0–28 mm), and the median distance from the LSA to the primary entry tear measured 31 mm (8–84 mm). Most of the patients had a type 2 or type 3 aortic arch anatomy with 47.2% and 43.8%, respectively.

### 3.2. Suitability

#### 3.2.1. Castor

The Castor as a CMD was anatomically suitable in 82% (95% CI 73.1 to 89.0%) of the patients using a total of 74 different configurations. The primary reasons for anatomical unsuitability were a too short proximal landing zone (n = 9), excessive kinking of the LSA (n = 3), an inappropriate arch geometry (n = 2), or a separate vertebral branch between the LSA and LCCA (n = 2). In one patient, the Castor device was unsuitable due to a narrow distal aorta; in another patient, implantation of the endoprosthesis was unfeasible because of a shaggy aorta.

When comparing the anatomical measurement results of patients deemed suitable for treatment with the CMD Castor device and those considered unsuitable, a significant difference was only observed regarding the distance between the LCCA and LSA (11.0 (8.0–14.2) vs. 8.0 (7.0–12.0), *p* = 0.047) (Table 4).

#### 3.2.2. TBE

In the same patient cohort, the off-the-shelf suitability of the TBE was 22% (14.3–31.4%). The reasons for TBE unsuitability were consistent with those for the Castor CMD graft. However, the primary anatomical reason for TBE unsuitability was an aortic diameter that was too small at the intended distal landing zone (Ishimaru zone 4), observed in 46 patients. Another ten patients had a too large distal aortic diameter. Lastly, four patients were deemed unsuitable due to an insufficient proximal landing zone length which would result in (partial) covering of the LCCA by the TBE main body. Table 5 shows that unsuitable patients had a 4 mm difference between proximal and distal aortic diameters (Ishimaru zones 2 and 4), while suitable patients had similar aortic diameters in these zones.

### 3.3. Optimal Off-the Shelf-Stock

#### 3.3.1. Castor—Optimal Off-the-Shelf Stock

**Figure 3:**
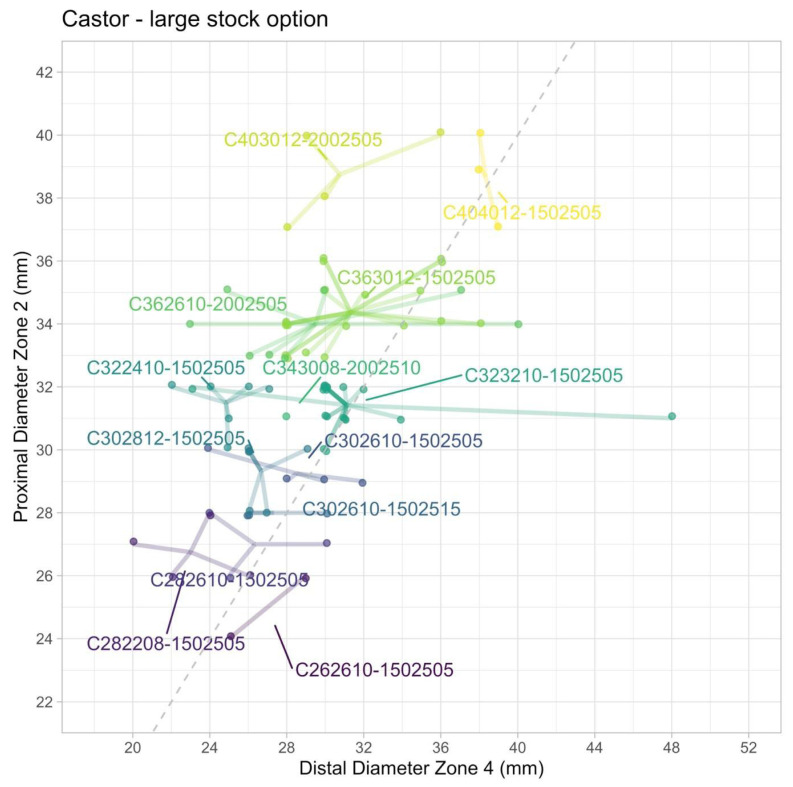
Castor Unibody single branch TEVAR as an off-the-shelf solution, showing 13 optimal configurations to treat 82% of the patients with acute TBAD, when additional procedures such as branch or TEVAR extension is allowed.

Figure 3 shows the centroids for optimal graft configurations to treat all 82 patients that are anatomically suitable for a Castor device. With 13 distinct graft configurations 34 of the 82 patients can be treated without any adjunctive procedures, resulting in an off-the-shelf suitability of 34% (24.8–44.2%). Of note, 10 of these 13 configurations are tapered.

By allowing adjunctive procedures—such as distal aortic stent graft extensions in both standard and reversed trombone configurations, and extending the LSA branch—all the 82 anatomically suitable patients can be treated with the Castor device with only these 13 configurations. Adjunctive procedures mainly included LSA stent graft extensions (n = 21), followed by distal aortic stent graft extensions (n = 19), or both (n = 8) (Table S1).

#### 3.3.2. TBE—Optimal Off-the-Shelf Stock

**Figure 4:**
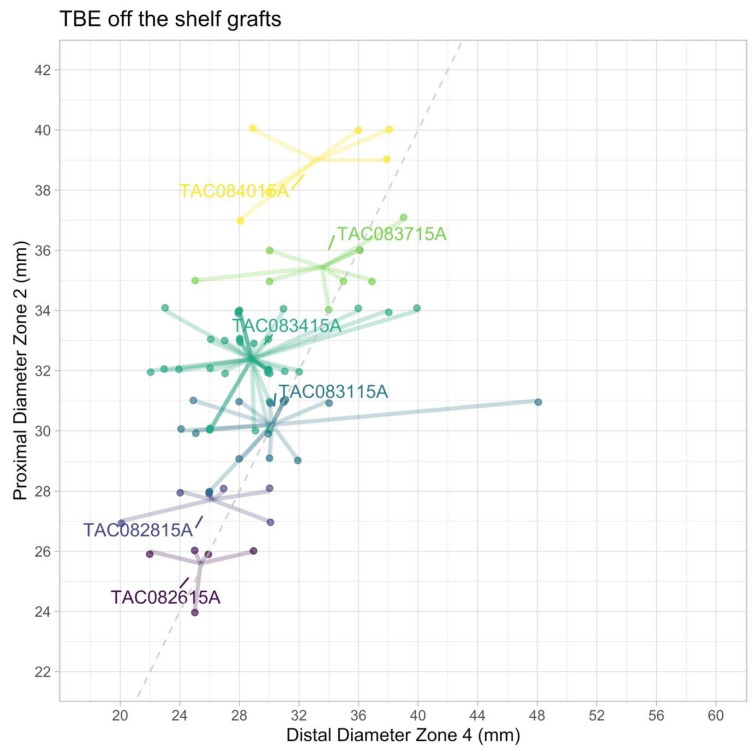
Thoracic Branch Endoprosthesis (TBE) with a reduced optimized off-the-shelf solution, showing six optimal configurations to treat 78% of the patients with acute TBAD, when additional procedures such as branch or TEVAR extension is allowed.

The optimal off-the-shelf stock for the GORE platform included six distinct main grafts (out of the 26 available options) with five different LSA branches, reaching a suitability of 78% (68.6–85.7%) when adjunctive procedures are allowed. Of note, all six main grafts had an 8 mm portal option and were 15 cm in length (Table S1). However, additional TEVAR implantation would be needed in 56 patients (Figure 4, Table S2). In most of these cases (46 out of 56), aortic stent graft extension in reversed trombone technique is needed to avoid extensive oversizing of the TBE in the smaller distal aorta.

### 3.4. Comparison Off-the-Shelf Suitability and Proximal Sealing

**Figure 5:**
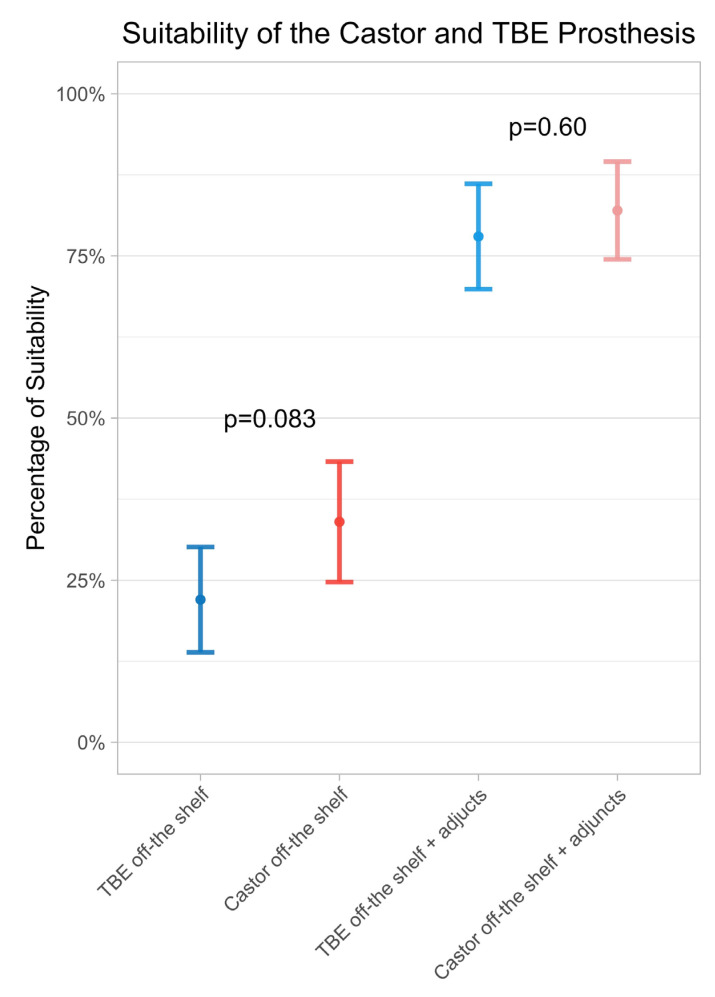
Comparison of the suitability of Castor and TBE, firstly if both were only available in an off-the-shelf version and secondly if additional procedures such as distal extension or branch extension are permitted.

The off-the-shelf suitability without adjunctive procedures was 34% for the Castor graft, which required 13 distinct configurations, compared to 22% for the TBE graft, which involved six main graft and five side branch configurations. This difference was not statistically significant (*p* = 0.083). If adjunctive procedures were allowed, the off-the-shelf suitable increased to 82% for the Castor and to 78% for the TBE device, without statistically significant difference (*p* = 0.60), (Figure 5).

**Figure 6:**
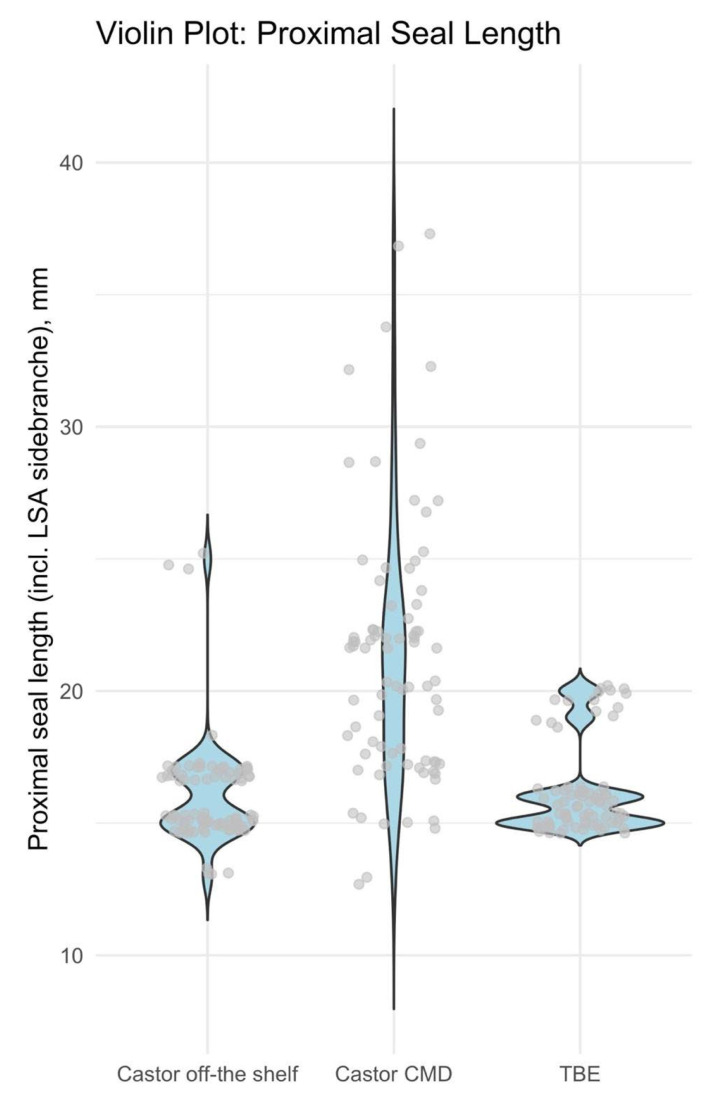
Violin Plot for comparison of the proximal sealing length (covered graft lengths from the proximal edge of the graft to the distal end of the portal (TBE length) or the distal end of the branch (Castor)) of both single-branched thoracic endografts.

The longest proximal sealing length was achieved with the Castor CMD solution, with a median of 22 mm (q1–q3: 17–23 mm), in the treatment of all 82 patients. This was significantly longer than the sealing lengths achieved with the Castor off-the-shelf graft (median 15 mm, q1–q3: 15–17 mm) and the TBE (median 16 mm, q1–q3: 15–16 mm), which treated 78 patients; *p* < 0.001 for both comparisons. There was no significant difference in proximal sealing length between the two off-the-shelf solutions (*p* = 0.638) (see Figure 6).

## 4. Discussion

The findings of this study will provide crucial insights into the anatomical feasibility and optimization of off-the-shelf stocks for two commercially available single-branched TEVARs—the Castor and the Gore TBE—in the management of acute TBAD. To the best of our knowledge, this is the largest analysis focusing specifically on acute TBAD patients, a cohort in which timely therapy is critical and off-the-shelf solutions are highly desirable. In the setting of acute TBAD, the time required for manufacturing custom-made devices—often several weeks—limits their applicability, making off-the-shelf solutions essential for timely treatment in patients with acute or complicated presentations.

The main finding is that the anatomy of most acute TBAD patients can be accommodated by a single-branched device landing in zone 2, especially when adjunctive stent grafts are available to extend the coverage length. Approximately, four out of five patients (80%) could be treated with either device. Additionally, the analysis informs what an off-the-shelf device inventory should involve: for instance, the most common aortic diameters were around 32–38 mm. Overall, the most common anatomical limitations for off-the-shelf use were insufficient proximal sealing length in zone 2, pronounced distal aortic tapering causing distal diameter mismatch, and unfavorable left subclavian artery anatomy.

These findings reinforce and extend prior work on branched arch devices. Leone et al. examined the Castor device in a mixed population of arch pathologies (dissections, aneurysms, penetrating aortic ulcers) and found 68.1% of anatomical feasibility [13]. Our higher rate of 82% for TBAD patients may be due to the fact that dissections—particularly acute ones—may have more favorable anatomy for such devices than chronic degenerative aneurysms. Dissections often occur in vessels that are not massively dilated. In our cohort, the median proximal aortic diameter was 32 mm, which is well within the device range [20]. There is also less arch tortuosity. Moreover, we examined acute dissections in which the aorta has not yet undergone chronic remodeling. This could result in a more uniform population. However, one must consider that some anatomies could deteriorate (e.g., false lumen expansion/aneurysm formation) over time. The discrepancy may also be due to different inclusion criteria. Leone et al. required that all their TEVAR cases be performed in zone 2. In contrast, we hypothetically placed devices in patients who were managed with best medical therapy only, since we included uncomplicated TBAD as well. However, the results were similar in terms of the number of configurations in the off-the-shelf Castor configuration: 13 in our study and 21 in Leone’s results, although these could be reduced to 12 if slightly less stringent criteria were applied [13].

In the case of TBE, our analysis also aligns with previous reports. Magee et al. first highlighted the stringent anatomical criteria, with only 28% of acute TBADs meeting all requirements—essentially matching our initial 22% suitability [14]. This low percentage could be misconstrued as meaning the device is not useful for many patients. However, our study shows that the applicability increases considerably if one is willing to add one or two additional stent grafts. The group around Leone also investigated the TBE and recently published their results. They demonstrated that the TBE is suitable for 76% of patients with mixed aortic pathologies (50% dissections, n = 46), a number that can rise to 84% with an “extended” definition of suitability that allows for LSA solutions. They also emphasized that 87% of suitable patients could be treated with five aortic components and that four branch sizes fit 89% of cases, which is very similar to our findings [15].

The main advantage of the TBE seems to be the flexibility in choosing different LSA components. This makes the TBE solution, with six main body and five branch configurations, more efficient than the Castor solution, which has 13 configurations. However, the off-the-shelf Castor solution clearly outperforms the TBE solution in terms of the number of additional procedures required for distal aortic stent graft extensions (27 versus 56). Besides the financial aspect, this can be advantageous when limited coverage is preferred. On the other hand, the risk of expiration of 13 distinct Castor grafts must be considered when holding a stock of them. This highlights a key point for hospital systems: fewer devices can be stocked, but more adjunct components must be used per case, and sizing may be slightly suboptimal. A local optimal middle ground likely exists, depending on case volume, specific reimbursement policy, and commercial availability of the grafts.

Another main finding this study highlights is, that the aortic diameter tapers from zone 2 to zones 4–5 in many patients. This creates a challenge because if a graft is chosen to fit proximally, it may be oversized distally by more than the recommended 0–10%. Tapered devices are therefore very helpful for avoiding oversizing in the distal aorta. Alternatively, implantation of a smaller distal thoracic stent graft in reversed trombone can be used. In some cases, tapering can be so extreme that patients require a tapered stent graft to achieve the desired 10% of oversizing. The TBE, in its current iteration, has a uniformly sized main trunk. Thus, in TBAD where tapering is significant, having tapered graft options in the inventory would be favorable. A tapered single-branch device design with a smaller distal diameter would likely reduce the need for distal adjuncts in dissections.

To accommodate the widest range of patients, the Castor configurations were planned with relatively small LSA branch diameters (8, 10, or 12 mm maximum) and the shortest available branch length (25 mm). This approach eliminates extensive oversizing but it may result in potentially undersized side branches for some patients. With the shortest branch length, extension with a balloon-expandable covered stent can be used to achieve adequate sealing, if necessary, without encroaching on the vertebral artery ostium. In some cases, leaving a slightly undersized branch may be acceptable if the primary entry tear is effectively sealed by the main graft and no residual false lumen persists. Nonetheless, the modular branch design of the TBE offers greater flexibility from a technical standpoint, which translates into fewer LSA adjunctive procedures. Conversely, the unibody design of the Castor graft eliminates the risk of stent graft dislocation. Furthermore, it eliminates the need for antegrade advancement of the side branch through a portal into the LSA, a technically challenging step during TBE deployment.

A clear advantage of ordering a custom-made Castor stent graft is the ability to tailor the device to achieve an optimal proximal sealing length. This study demonstrated that the proximal sealing length was significantly longer with the CMD solution than with the off-the-shelf TBE or Castor stent grafts. To maximize anatomical compatibility with off-the-shelf Castor stent grafts, the covered length proximal to the LSA branch was reduced to 5 mm. While this adaptation increased suitability across a broader patient population with fewer configurations needed, it resulted in a shorter proximal seal. Consequently, the sealing advantage of the CMD version was lost and thus, the benefit of a longer proximal seal of the Castor CMD was lost.

### 4.1. Clinical Applications

The findings of this study have direct implications for clinical decision-making in the management of acute TBAD. First, our results support the feasibility of using off-the-shelf single-branched endovascular devices in the majority of patients requiring zone 2 landing, enabling timely intervention in a clinical setting where treatment delays may adversely affect outcomes. Second, the anatomical distribution of aortic diameters and branch configurations identified in this cohort can inform rational stock planning, allowing centers to optimize device availability while minimizing inventory burden.

Furthermore, the comparative analysis of Castor and TBE configurations highlights trade-offs between device modularity, need for adjunctive components, and sealing strategies, which may guide individualized device selection based on patient anatomy, institutional experience, and logistical constraints. Finally, recognition of frequent distal aortic tapering underscores the importance of tapered graft options or staged distal extension strategies to avoid excessive oversizing, particularly in acute dissections.

### 4.2. Future Directions

As these devices become more broadly available, the next logical step will be the implementation of prospective clinical studies. Future investigations of acute aortic dissections should not only assess anatomical feasibility and technical success, but also systematically evaluate clinically relevant outcomes such as procedural success, perioperative complications—particularly stroke rates—and long-term aortic remodeling.

In addition, the potential of branched endovascular devices to promote more complete false lumen thrombosis in the aortic arch and descending aorta by sealing the primary entry tear while maintaining perfusion to vital supra-aortic branches warrants further investigation. While this concept is promising, it remains hypothetical and must be confirmed in adequately powered clinical studies.

Finally, future research should incorporate formal health economic analyses and structured assessments of procedural duration, resource utilization, and perioperative logistics. In the present study, any potential economic or operational implications are theoretical and were not quantified, highlighting an important area for future investigation.

### 4.3. Limitations

This study has several limitations that should be considered. As a single-center retrospective analysis with a relatively small sample size—despite being the largest study investigating this topic with acute TBAD—the strength and generalizability are limited. Although the study spans a long inclusion period, there is currently no evidence suggesting a temporal change in the anatomical morphology of acute type B aortic dissections; however Additionally, the analysis is based purely on imaging and hypothetical device deployment. These patients were not treated with branched devices, as these devices were not commercially available during most of the study period. Thus, we assume perfect deployment and tolerance of certain anatomical criteria that, in practice, have some margin. On the other hand, our criteria attempted to address obvious issues (e.g., extreme arch tortuosity). Furthermore, were measurements derived from static CTA images in accordance with current standards; thus, dynamic changes in the true lumen caused by the dissection flap could not be assessed, representing an inherent limitation of CTA-based morphometric analyses. Another limitation is that we only considered cases with required proximal landing in zone 2 and LSA debranching. A few patients had such short proximal landing zones that zone 2 was not sufficient and landing in zone 1 or even 0 with >2 branches was necessary. We simply counted those patients as unsuitable. As multi-branched arch devices evolve, some TBADs that are currently unsuitable for a single branch may become treatable with more complex devices or in combination with open debranching methods. However, this was beyond the scope of our study., subtle trends cannot be completely excluded.

## 5. Conclusions

In summary, both single-branched thoracic aortic stent grafts are promising options for extending the proximal landing zone when treating acute TBAD. With additional procedures, mainly distal extensions, they have an overall suitability rate of up to 80%. For TBAD, a tapered design is preferred. However, a reduced stock of both systems requires a higher rate of additional procedures, which must also be considered in terms of cost.

## Figures and Tables

**Table 1:** Comparison of the two single-branch platforms based on the manufacturer’s specifications. TBE = Thoracic Branch Endoprosthesis; LSA = left subclavian artery.

	Castor	TBE
Design type	Unibody	Bi-Modular
Branch type	LSA branch incorporated in Unibody	Single inner-branch for LSA
Availability	Custom-made	Off-the-shelf
Sizes of the main body (diameter and length)	26–44 mm 6–21 cm	21–45 mm 10, 15, 20 cm
Branch sizes (diameter)	6–14 mm	8–20 mm
Tapering	Possible	Not possible
Possible number of configurations	351,000 possible configurations	110 possible configurations: 26 different main grafts: 16 × 8 mm Portal and 10 × 12 mm Portal 8 different branch sizes: 5 for the 8 mm portal and 3 for the 12 mm portal)
Market availability	Approved in China and CE-marked	FDA-approved (U.S.) and CE-marked
Delivery system	24 F	20–26 F
Proximal extension	Not available	8 different proximal Extensions: diameter 21–45 mm, length 3.6–4.6 cm

**Table 3:** Patient and anatomical characteristics.

Total (*n* = 100)	
Patient characteristics	
Age in years, median (Q1, Q3)	63.0 (55.1, 71.3)
Male Sex	75 (75%)
BMI, median (Q1, Q3)	26.1 (23.5, 30.1)
Hypertension	78 (78%)
Diabetes	11 (11.2%)
Smoking	40 (40.0%)
COPD	11 (11%)
Coronary heart disease	31 (31%)
Dyslipidemia	18 (18%)
Chronic kidney disease	23 (23%)
**Anatomical characteristics**	
Max proximal aortic diameter, mm	32 (30, 34)
Max distal aortic diameter, mm	30 (26, 31)
Max LSA diameter, mm	11 (9, 12)
LCCA to LSA length, mm	10 (8, 14)
LSA to dissection length, mm	3 (0, 28)
LSA to primary entry tear length, mm	31 (8, 84)
Iliac access not suitable	1% (1.2%)
**Aortic arch type**	
Type 1	8% (9.0%)
Type 2	42% (47.2%)
Type 3	39% (43.8%)

**Table 4:** Anatomical comparison between patients deemed suitable for treatment with the custom-made Castor device and those considered unsuitable. LCCA = left common carotid artery, LSA = Left subclavian artery.

Variable, mm, Median (Q1, Q3)	Suitable (n = 82)	Unsuitable (n = 18)	*p*-Value
**Proximal aortic diameter**	32.0 (30.0, 34.0)	31.0 (26.0, 36.0)	0.848
**Distal aortic diameter**	29.5 (26.0, 32.0)	28.0 (24.5, 31.5)	0.110
**LSA distal diameter**	11.0 (10.0, 12.0)	10.5 (8.8, 11.5)	0.404
**LCCA to LSA length**	11.0 (8.0, 14.2)	8.0 (7.0, 12.0)	0.047
**LSA to dissection length**	6.0 (0.0, 31.5)	0.0 (0.0, 11.5)	0.463
**LSA to primary entry tear length**	38.0 (10.0, 95.5)	15.0 (0.0, 52.5)	0.491

**Table 5:** Anatomical comparison between patients deemed suitable for treatment with the off-the-shelf Thoracic Branch Endoprosthesis (TBE) and those considered unsuitable. LCCA = left common carotid artery, LSA = left subclavian artery.

Variable, mm, median (Q1, Q3)	Suitable (n = 22)	Unsuitable (n = 78)	*p*-Value
**Proximal aortic diameter**	31 (28, 34)	32 (30, 34)	0.508
**Distal aortic diameter**	31 (28, 34)	28 (26, 30)	0.045
**LSA distal diameter**	11 (9, 12)	11 (9, 12)	0.589
**LCCA to LSA length**	10 (8, 15)	10 (8, 13)	0.692
**LSA to dissection length**	12 (0, 25)	2 (0, 28)	0.940
**LSA to primary entry length**	40 (30, 67)	25 (7, 90)	0.644

## Data Availability

The datasets generated during and analyzed during the current study are available from the corresponding author on reasonable request.

## Supplementary Materials

The following supporting information can be downloaded at: https://www.mdpi.com/article/10.3390/jcm15020558/s1, Table S1: Optimal off-the-shelf stock for the Endovastec Castor Unibody-Stent graft, as well as for the Gore Thoracic Branch Endoprosthesis (TBE) main body as well as branch; Table S2: Size and frequency of TEVAR extensions in the reduced optimal TBE of the shelf solution.

## Abbreviations

The following abbreviations are used in this manuscript:

CMD	custom-made device
CTA	Computer-tomography angiography
LCCA	Left common carotid artery
LSA	Left subclavian artery
TBAD	Acute Type B aortic dissection
TBE	Thoracic Branch Endoprosthesis
TEVAR	Thoracic endovascular aortic repair

## References

[1] Perone F., Guglielmo M., Coceani M., La Mura L., Dentamaro I., Sabatino J., Gimelli A. (2023). The Role of Multimodality Imaging Approach in Acute Aortic Syndromes: Diagnosis, Complications, and Clinical Management. Diagnostics.

[2] Riambau V., Böckler D., Brunkwall J., Cao P., Chiesa R., Coppi G., Czerny M., Fraedrich G., Haulon S., Jacobs M. (2017). Editor’s Choice—Management of Descending Thoracic Aorta Diseases. Eur. J. Vasc. Endovasc. Surg..

[3] Oberhuber A., Maßmann A., Betge S., Raddatz A., Ott C., Ploenes C., Ito W., Janosi R.A., Langheim E., Czerny M. Leitlinie—S2K Typ B Aortendissektion. https://register.awmf.org/assets/guidelines/004-034l_S2k_Typ_B_Aortendissektion_2022-05.pdf.

[4] MacGillivray T.E., Gleason T.G., Patel H.J., Aldea G.S., Bavaria J.E., Beaver T.M. (2022). The Society of Thoracic Surgeons/American Association for Thoracic Surgery Clinical Practice Guidelines on the Management of Type B Aortic Dissection. Ann. Thorac. Surg..

[5] Mazzolai L., Teixido-Tura G., Lanzi S., Boc V., Bossone E., Brodmann M. (2024). 2024 ESC Guidelines for the management of peripheral arterial and aortic diseases: Developed by the task force on the management of peripheral arterial and aortic diseases of the European Society of Cardiology (ESC) Endorsed by the European Association for Cardio-Thoracic Surgery (EACTS), the European Reference Network on Rare Multisystemic Vascular Diseases (VASCERN), and the European Society of Vascular Medicine (ESVM). Eur. Heart J..

[6] Mesar T., Alie-Cusson F.S., Rathore A., Dexter D.J., Stokes G.K., Panneton J.M. (2021). A more proximal landing zone is preferred for thoracic endovascular repair of acute type B aortic dissections. J. Vasc. Surg..

[7] Dueppers P., Meuli L., Reutersberg B., Hofmann M., Messmer F., Zimmermann A. (2021). Early and Mid-Term Outcomes of Open versus Endovascular Left Subclavian Artery Debranching for Thoracic Aortic Diseases. Ann. Thorac. Cardiovasc. Surg..

[8] Feezor R.J., Lee W.A. (2009). Management of the Left Subclavian Artery during TEVAR. Semin. Vasc. Surg..

[9] Garg K., Maldonado T.S. (2012). Further consideration for subclavian revascularization with TEVAR. Semin. Vasc. Surg..

[10] Ye P., Miao H., Zeng Q., Chen Y. (2024). Comparison of total percutaneous in situ microneedle puncture and chimney technique for left subclavian artery fenestration in thoracic endovascular aortic repair for type B aortic dissection. Eur. Radiol..

[11] Brusa J., Lutz E., Schoenhoff F.S., Weiss S., Schmidli J., Makaloski V. (2022). One-Year Outcome of Postoperative Stroke and Nerve Injury After Supraclavicular Revascularization of The Left Subclavian Artery for Proximal Landing Zone Extension in Thoracic Endovascular Aortic Repair. Ann. Vasc. Surg..

[12] Huang Q., Chen X.M., Yang H., Lin Q.N., Qin X. (2018). Effect of Left Subclavian Artery Revascularisation in Thoracic Endovascular Aortic Repair: A Systematic Review and Meta-analysis. Eur. J. Vasc. Endovasc. Surg..

[13] Leone N., Andreoli F., Bartolotti L.A.M., Migliari M., Baresi G.F., Saitta G., Silingardi R., Gennai S. (2023). Anatomical feasibility of a ‘semi-custom’ unibody single-branch endograft in previous zone 2 thoracic endovascular aortic repair. Eur. J. Cardiothorac. Surg..

[14] Magee G.A., Veranyan N., Kuo E.C., Ham S.W., Ziegler K.R., Weaver F.A., Fleischman F., Bowdish M.E., Han S.M. (2019). Anatomic suitability for “off-the-shelf” thoracic single side-branched endograft in patients with type B aortic dissection. J. Vasc. Surg..

[15] Leone N., Andreoli F., Bartolotti L.A.M., Ferri A., Silingardi R., Gennai S. (2025). Anatomical Suitability of a Standard Subclavian Branched Endograft in Previous Zone 2 Thoracic Endovascular Aortic Repair. J. Endovasc. Ther..

[16] Yoon W.J., Mani K., Wanhainen A., Rodriguez V.M., Mell M.W. (2021). Anatomic feasibility of off-the-shelf thoracic single side-branched endograft in patients with blunt traumatic thoracic aortic injury. J. Vasc. Surg..

[17] Marrocco-Trischitta M.M., de Beaufort H.W., Secchi F., van Bakel T.M., Ranucci M., van Herwaarden J.A., Moll F.L., Trimarchi S. (2017). A geometric reappraisal of proximal landing zones for thoracic endovascular aortic repair according to aortic arch types. J. Vasc. Surg..

[18] Rizza A., Trimarchi G., Di Sibio S., Bastiani L., Murzi M., Palmieri C., Foffa I., Berti S. (2023). Preliminary Outcomes of Zone 2 Thoracic Endovascular Aortic Repair Using Castor Single-Branched Stent Grafts: A Single-Center Experience. J. Clin. Med..

[19] Fang C., Wang C., Liu K., Pang X. (2021). Early Outcomes of Left Subclavian Artery Revascularization Using Castor Single-Branched Stent-Graft in the Treatment of Type B Aortic Dissection or Intramural Hematoma. Ann. Thorac. Cardiovasc. Surg..

[20] Paratz E.D., Nadel J., Humphries J., Rowe S., Fahy L., La Gerche A., Prior D., Celermajer D., Strange G., Playford D. (2024). The aortic paradox: A nationwide analysis of 523 994 individual echocardiograms exploring fatal aortic dissection. Eur. Heart J. Cardiovasc. Imaging.

