# Supplementary material for: Suitability of Single-Branched Thoracic Endografts for the Treatment of Acute Type B Aortic Dissection—An Anatomical Feasibility and Comparative Study
Source: J Clin Med. 2026 Jan 9;15(2):558. doi: 10.3390/jcm15020558 (PMC12842510; doi:10.3390/jcm15020558)
Supplement: Supplementary file 1 [file jcm-15-00558-s001.zip › jcm-4062894-supplementary.pdf]

Table S1. Optimal off-the-shelf stock for the Endovastec Castor Unibody-Stent graft, as well as for the Gore Thoracic Branch Endoprosthesis (TBE) main body as well as branch

**Castor off-the-shelf stock**	**TBE reduced off-the-shelf stock (Main body)**	**TBE reduced off-the-shelf stock (Branch)**
C403012-2002505	TAC084015A	TSB080806A
C404012-1502505	TAC083715A	TSB081006A
C363012-1502505	TAC083415A	TSB081206A
C362610-2002505	TAC083115A	TSB081506A
C343008-2002510	TAC082815A	TSB081706A
C323210-1502505	TAC082615A	
C302610-1502505		
C302812-1502505		
C302610-1502505		
C302610-1502515		
C282610-1502505		
C282208-1502505		
C262610-1502505		

Suppl. table 1. Optimal off-the-shelf stock for the Endovastec Castor Unibody-Stent graft, as well as for the Gore Thoracic Branch Endoprosthesis (TBE) main body as well as branch

Table S2. Size and frequency of TEVAR extensions in the reduced optimal TBE of the shelf solution.

**Gore cTAG distal extension**	**Frequency of Necessity**
TGM262110E	4x
TGM262610E	5x
TGM282810E	17 x
TGM312610E	2x
TGM313110E	20x
TGM343410E	1x
TGM373710E	3x
TGM404010E	4x

Suppl. table 2.
